# Light response and adsorption interaction of black phosphorus quantum dots and single-layer graphene phototransistor

**DOI:** 10.1007/s12200-023-00065-4

**Published:** 2023-05-24

**Authors:** Qi Han, Yadong Jiang, Xianchao Liu, Chaoyi Zhang, Jun Wang

**Affiliations:** 1 School of Optoelectronic Science and Engineering, University of Electronic Science and Technology of China, Chengdu, 610054 China; 2 State Key Laboratory of Electronic Thin Films and Integrated Devices, University of Electronic Science and Technology of China, Chengdu, 610054 China

**Keywords:** Black phosphorus quantum dots, Dirac point, Photoconduction, Adsoprtion

## Abstract

Black phosphorus quantum dots (BPQDs) are synthesized and combined with graphene sheet. The fabricated BPQDs/graphene devices are capable of detecting visible and near infrared radiation. The adsorption effect of BPQDs in graphene is clarified by the relationship of the photocurrent and the shift of the Dirac point with different substrate. The Dirac point moves toward a neutral point under illumination with both SiO_2_/Si and Si_3_N_4_/Si substrates, indicating an anti-doped feature of photo-excitation. To our knowledge, this provides the first observation of photoresist induced photocurrent in such systems. Without the influence of the photoresist the device can respond to infrared light up to 980 nm wavelength in vacuum in a cryostat, in which the photocurrent is positive and photoconduction effect is believed to dominate the photocurrent. Finally, the adsorption effect is modeled using a first-principle method to give a picture of charge transfer and orbital contribution in the interaction of phosphorus atoms and single-layer graphene.

**Graphical Abstract:**

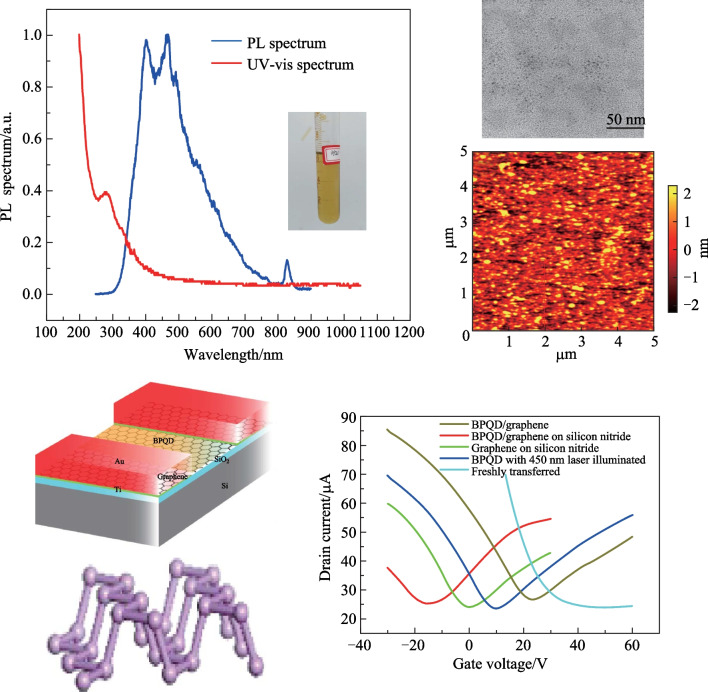

## Introduction

Black phosphorus (BP) is a direct bandgap semiconductor with a bandgap ranging from 0.3 eV for bulk material to 1.5 eV for a monolayer. The geometry dependent energy band gap and high absorption make BP important in optoelectronic detection, especially in near-infrared frequencies [1]. Anisotropy is an essential characteristic ingrained in the crystal alignment and energy band structure of BP, and it can be also observed in electrical and optical experiments [2]. The bandgap of thin layer BP is recognized to change with sample thickness, and photodetectors based on BP heterostructure have been reported [3, 4]; however, thin layer BP is rather difficult to be obtained due to lacking an available large-area fabrication method. Potential application of BP is severely constricted by its low production efficiency. Furthermore, thin film BP undergoes degradation in an aerobic environment and usually needs special protection such as by spin-coating a layer of high polymer. The solvothermal synthetization of black phosphorus quantum dots (BPQDs) proposed by Ren et al. [5] and Liu et al. [6], provides a promising approach for the application of BP.

Graphene is a kind of two-dimensional material with good conductivity, but the low absorption coefficient limits its application in photodetectors. Some methods can improve the performance of photodetectors, such as plasma modification, lead substitution, heterostructure formation and thickness control of the active material [7–10]. Among these methods, quantum dot technology offers special spectral features to conventional materials in the optical frequencies. The quantum confinement effect enhances the absorption and irradiance efficiency [11] in a certain narrow wavelength range, enabling high selectivity spectral filtering for detection or display applications [12]. For the case of detection, the multiexciton effect in quantum dots allows multiple pairs of excited carriers to be generated by one single photon, thus the quantum efficiency is increased several times when the energy of the incident photon is higher than twice the bandgap of the active material [13]. One advantage of quantum dots is that their bandgap can be tuned by varying their size.

BPQD show saturable absorption characteristic and this can be exploited in laser mode locking [14]. The photon-harvesting ability of quantum dots can be applied in photodetectors [6].

In this work, a comprehensive investigation of the adsorption and related doping effect of BPQDs on graphene is presented. Phototransistors based on graphene or BPQDs/graphene are fabricated. The doping effect is confirmed by manipulating the Dirac point both in dark mode and under illumination using different substrates. To fully identify the n-doping effect of BPQDs, an intrinsic graphene is needed. A Si_3_N_4_/Si substrate is adopted and processed with bufferred oxide etch (BOE) to achieve a nearly intrinsic graphene layer. Expectedly, the Dirac point shifts to the negative side after the BPQDs are applied. Photoresist induced photocurrent on the surface of graphene is first reported in this work. A new structure is designed to remove the effect of photoresist on graphene leaving only the effect of BPQDs in the experiment. Photocurrent rises rapidly when the device operates under vacuum conditions in a cryostat. The mechanism for photosensitivity in the cryostat is believed to be photoconductivity, and totally different from the mechanism at room temperature, where unintentional doping of graphene is compensatedby BPQDs. An ab initio simulation of the BPQDs/graphene system is carried out to present the energy band unfolding, charge transfer and orbital contribution. The phosphorous atom acts as an electron donor and the net charge transfer is − 0.294 e.

## Materials and methods

BPQD are fabricated by means of liquid exfoliation introduced by Zhang’s group [5]. The ratio of mass of bulk BP to the volume of N-methyl-2-pyrrolidone (NMP) liquid is 1 mg:1 mL. Photoluminescence (PL) spectra and absorption spectra are obtained for the diluted suspension. The PL spectra are observed and recorded by Hitachi Fluorescence F-4600 and the UV–visible spectra are measured by Shimadzu UV-1700 spectrophotometer. The AFM graph is taken by Ntegra Prima from NT-MDT. The transmission electron microscopy (TEM) image is obtained by a transmission electron microscope Tecnai G2 F20 from Field Electron and Ion Company with an acceleration potential difference of 100 kV.

The structure of the phototransistor is shown in the inset of Fig. 2a. The monolayer graphene is synthesized by chemical vapor deposition (CVD) on copper foil and transferred onto a highly p-doped Si substrate, covered by a 300 nm SiO_2_ layer utilizing thermal oxidation or a 50 nm Si_3_N_4_ layer utilizing conventional wet method [15]. Then a lift-off procedure starts to define the electrodes on graphene. Source and drain electrodes are defined through UV photolithography using a URE-2000 photolithography machine and a negative tone resist of NR9-3000PY, leaving a channel with a fixed width $W=600\; \mathrm{ \mu m}$ and length $L$ ranging from 20 to 600 μm. A layer of Au (120 nm)/Ti (5 nm) is adopted for metallization using electronic beam evaporation. A second UV lithography process is then conducted to protect graphene in the channel from oxygen plasma etching. Then a GaSonics AURA 1000 Stripper is used to remove the redundant graphene at $100\mathrm{^\circ{\rm C} }$ for 60 s. The BPQDs-NMP suspension liquid as prepared is diluted 20 times to prevent BPQDs from coupling or pilling up on the graphene sheet, which would result in a serious heating effect and interfere with the electro-optical response. The graphene sheet is drop-casted with the BPQDs liquid and let it stand for 1 min. Whereafter, the NMP solvent is removed by filter paper gently and the graphene sheet is then heated on a hot plate ($100\mathrm{^\circ{\rm C} }$, 10 s) for further liquid evaporation. To remove the effect of the photoresist, a metal mask is employed instead of photolithography. The device is measured in ambient environment then transferred in to a cryostat, where the measurement can be conducted at room temperature or low-temperature with vacuum condition in. Electrical measurement is performed by a Keysight 2636B source meter equipped with PRCBE mini probing station. The laser is powered by a direct current source controlled by a thyristor chopper circuits.

## Results and discussion

**Fig. 1:**
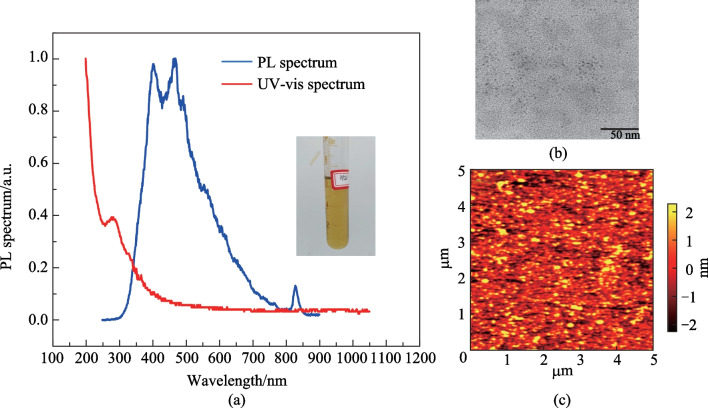
Spectrum and surface topography of BPQDs. **a** Photoluminescence spectrum and ultraviolet (UV)-visible spectra of BPQDs in NMP solvent. The inset shows a photograph of the transparent BPQDs turbid liquid. **b** TEM image of BPQDs. The sample is prepared by dropping the BPQDs turbid liquid on a copper mesh to provide enough contrast under TEM. The heavy dark regions are the conglomerate BP clusters. **c** AFM image of BPQDs/graphene surface

The synthesized BPQDs in NMP solvent is shown in the inset of Fig. 1a, and the corresponding photoluminescence (PL) and UV–visible absorption spectra are also illustrated. A sharp rise is observed in the ultraviolet wavelength range of the UV–visible absorption spectrum. The PL spectrum shows a main peak around 400 − 500 nm and a minor peak at 840 nm. The TEM image of BPQDs is obtained by dropping the BPQD liquid on copper mesh and removing NMP by evaporation. While the atomic force microscopy (AFM) image is taken for the BPQDs/graphene surface at the channel region of the phototransistor. The TEM image in Fig. 1b and AFM image in Fig. 1c provide intuitive information about the diameter and roughness of the BPQDs.

**Fig. 2:**
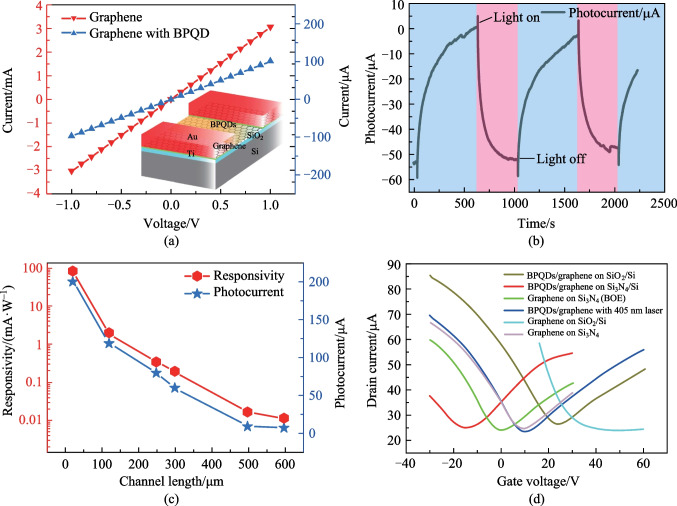
Photocurrent and responsivity of BPQDs/grapheme phototransistor. **a** current–voltage curves of graphene and BPQDs/graphene device. **b** Photocurrent of BPQDs/graphene device utilizing photolithography in the preparing process. Negative photocurrent is observed with a relatively long response time. The abrupt change of the photocurrent when light is switched on/off is due to the effect of photoresist on graphene. **c** Dependence of responsivity on channel length ranging from 20 to 600 μm with a bias of 0.1 V. **d** Transfer characteristic curve for the device with graphene or BPQDs/graphene on both SiO_2_/Si and Si_3_N_4_/Si substrate. The default substrate is SiO_2_/Si unless otherwise noted

A comparison of drain current ${I}_{\mathrm{d}}$ to drain-source voltage ${V}_{\mathrm{ds}}$ curves of a bare graphene device and a BPQDs/graphene device is illustrated in Fig. 2a. The resistance of bare graphene is 322 Ω and it increases to 649 Ω after the BPQDs are introduced. The photo-response curve fits a typical exponential feature $\Delta I={I}_{0}{\mathrm{e}}^{-t/\tau }$ with a time constant $\tau$ of 34.9 s as shown in Fig. 2b. The abrupt increase of the photocurrent at the instant of exposure to light is due to the interaction of negative tone photoresist and graphene, which will be further explained later.

The responsivity can be extracted the current–time curve. In Fig. 2b, a device with channel width of 600 μm and length of 30 μm is taken as an example. If the laser power is 7.6 mW, the responsivity reaches a value of 18.2 mA/W at a bias of 10 mV. The laser spot is estimated to be $0.5 \; {\mathrm{cm}}^{2}$ and is assumed to be uniform so the incoming power can be evaluated for a specific channel area. Noise equivalent power (NEP) is used to estimate the noise performance of a photodetector. Here, the noise current is evaluated to be 1.14 nA and the NEP is $1.6\times {10}^{-7 }\; \mathrm{W}$. The BPQDs/graphene device shows the highest responsivity to 405 nm laser. Devices with different channel length *L* are fabricated. For *L* ranging from 20 to 600 μm, the dependence of responsivity on channel length is shown in Fig. 2c. In a photoconductor, radiation produces excess electrons and holes with an intrinsic generation rate proportional to the product of the concentration of electrons and holes $G=\gamma {n}_{0}{p}_{0}$. The recombination rate $\gamma$ equals the generation rate at thermal equilibrium. Excess electrons recombine with majority holes at a rate of $R=rnp$, where *n* and *p* denote electron and hole concentration respectively. $n={n}_{0}+\Delta n,$ where $\Delta n$ is nonequilibrium electron concentrations. As such, the net electron recombination rate is given by ${U}_{\mathrm{d}}=R-G=\gamma \left({n}_{0}+{p}_{0}\right)\Delta n+\gamma {(\Delta n)}^{2}$. The responsivity can be approximated by the following equation when the concentration of photoexcited carriers is small [16]:

$$R=\frac{e\eta }{\sqrt{2}(hc/\lambda )}\frac{{\tau }_{\mathrm{trap}}}{{\tau }_{\mathrm{transit}}},$$

where $\eta$ is quantum efficiency, ${\tau }_{\mathrm{trap}}=\Delta n/{U}_{\mathrm{d}}=1/[\gamma ({n}_{0}+{p}_{0}+\Delta n)]$ is the trap-limited carrier lifetime, where $\upgamma$ represents the coefficient of electron–hole recombination, and ${\tau }_{\mathrm{transit}}={L}^{2}/(\mu {V}_{\mathrm{ds}})$ is carrier transit time in the channel, where $\mu$ is carrier mobility. Though this model does not fit our BPQDs/graphene system rigorously because the adsorption effect is important in ambient environment, the qualitative relationship between the responsivity and channel length or power of radiation is useful. As the channel length increases, ${\tau }_{\mathrm{transit}}$ also increases, eventually inducing a lower responsivity. Lifetime of the photoexcited electrons is inversely proportional to the summation of the intrinsic carrier concentration and the nonequilibrium electron concentration. As the power of the incident light increases, $\Delta n$ increases and ${\tau }_{\mathrm{trap}}$ decreases, eventually leading to a lower responsivity.

To find the origin of the photocurrent in the BPQDs/graphene system, transfer characteristic curve is measured and illustrated in Fig. 2d. Graphene sheet newly transferred onto the SiO_2_/Si and Si_3_N_4_/Si layer shows a natural p-doping due to the inevitable absorption of oxygen and water. A minimum conductivity is found around 60 V for SiO_2_/Si substrate, which is known as the Dirac point Fermi energy shift relative to the Dirac point can be calculated from the gate voltage at the Dirac point ${V}_{\mathrm{Dirac}}$ [17]:

$${E}_{\mathrm{f}}=\mathrm{\hslash }{v}_{\mathrm{F}}\sqrt{\uppi {C}_{\mathrm{ox}}({V}_{\mathrm{G}}-{V}_{\mathrm{Dirac}})/\mathrm{e}},$$

where ${v}_{\mathrm{F}}=1\times {10}^{6} \;\mathrm{m}/\mathrm{s}$ is the Fermi velocity, *V*_G_ is the gate voltage, and ${C}_{\mathrm{ox}}={\epsilon }_{\mathrm{ox}}/{t}_{\mathrm{ox}}$ is the capacitance per unit area of the insulator SiO_2_, with ${\epsilon }_{\mathrm{ox}}={\epsilon }_{\mathrm{r}}{\epsilon }_{0}$ representing the dielectric constant of the SiO_2_, ${t}_{\mathrm{ox}}$ defines the thickness of the dielectric. The dielectric constant of SiO_2_ is 3.9 and the thickness is 285 nm as determined by ellipsometry measurement. As a result, the Fermi energy shifts ${E}_{\mathrm{f}}=0.24\; \mathrm{ eV}$ downward relative to intrinsic condition. Electron and hole mobility of graphene can be obtained by using the transfer characteristic curve, and given by [18]:

$$\mu =\frac{L}{W{C}_{\mathrm{ox}}{V}_{\mathrm{ds}}}\times \frac{\partial {I}_{\mathrm{ds}}}{\partial {V}_{\mathrm{ds}}}.$$

The mobility is estimated to be ~$600\;  {\mathrm{cm}}^{2}/(\mathrm{V}\cdot \mathrm{s})$ for holes and $\sim 30\;  {\mathrm{cm}}^{2}/(\mathrm{V}\cdot \mathrm{s})$ for electrons with channel length $L=600\; \mathrm{ \mu m}$ and width $W=300\; \mathrm{ \mu m}$.

With the BPQDs on the graphene sheet, the Dirac point shift in the direction of negative axis. Accordingly, the graphene tends to be intrinsic. This shift can be understood if the adsorption of phosphorous atoms is taken into account. When graphene adsorbs oxygen or water, weak intermolecular bonds are formed between carbon atoms in graphene and oxygen or hydrogen atoms in the adsorbed molecule. Oxygen atoms possess higher attraction to the electron cloud, thus bare ion cores of carbon atoms are exposed, which is a necessary condition for p-doping. Phosphorous atoms have weaker electronegativity, with an electron affinity (${EA}_{\mathrm{phos}}$) = 0.746 eV [19, 20] compared to oxygen atoms (${EA}_{\mathrm{oxygen}}=1.47\; \mathrm{ eV}$) [21].

The work function of black phosphorous is difficult to determine because of various doping conditions and work function pinning effect. Due to the hydrophily of the insulating substrate material SiO_2_, graphene is unintentionally doped and this complicates the situation and makes it difficult to determine the additional doping effect of BPQDs. BOE, rinsing or substituting the dielectric material can reduce the doping concentration of graphene [22]. Si_3_N_4_ is used to serve as an insulating substrate and electrical measurement is again performed. As shown in Fig. 2d, ${V}_{\mathrm{Dirac}}$ locates around 7 V for graphene on Si_3_N_4_/Si. If the graphene is dealt with BOE, the Dirac point moves to zero. If the graphene on Si_3_N_4_/Si is drop casted with BPQDs, the adhesion of phosphorus atoms causes the ${V}_{\mathrm{Dirac}}$ to become negative and shift to − 15 V. In this process, intermolecular interaction contributes to the n-doping of graphene. In the case of BPQDs/graphene on SiO_2_ substrate, when laser radiation of 405 nm wavelength incidents to the device and BPQDs produces excess electrons and holes, the excess carriers transfer to graphene due to diffusion. These carriers balance out the p-doping effect of oxygen and water to graphene. The ${V}_{\mathrm{Dirac}}$ gradually moves towards negative axis with the laser illumination.

**Fig. 3:**
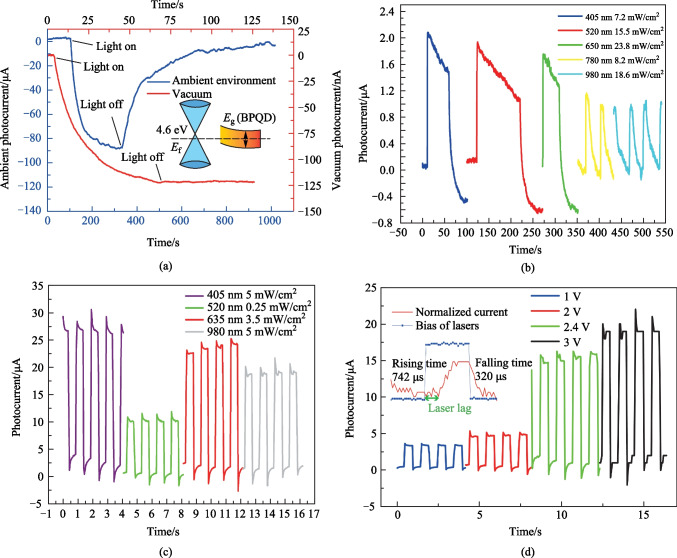
Photocurrent of the BPQDs/graphene device on SiO_2_/Si in a cryostat, exploring the origin of the photo-sensitivity mechanism. **a** Photocurrent measured in ambient air and vacuum respectively, for BPQDs/graphene device made by using metal mask. **b** Photocurrent measured in vacuum at low temperature of a BPQDs/graphene device made by using metal mask under laser radiation with wavelength ranging from 405 to 980 nm. **c** Photoresist-induced photocurrent for different radiation power and wavelength. **d** Photoresist-induced photocurrent under 980 nm laser radiation for various bias voltages. The inset shows the response speed of photoresist/graphene system under 980 nm laser radiation

The energy band of the BPQDs/graphene system under illumination can be modeled using the conventional energy band theory, in a similar way to modelling the PbS quantum dots/graphene hybrid system [23], as shown in the inset of Fig. 3a. For the case of BPQDs/graphene on SiO_2_ substrate, contact between BPQDs and graphene results in diffusion of carriers toward each other. The adsorbed oxygen and water are partly replaced by the BPQDs, relieving the p-doping of graphene. The density difference of holes and electrons in BPQDs is still less than that in graphene, thus holes tend to transfer from graphene to BPQDs and a built-in electric field pointing toward graphene is formed. Energy band then bend upward toward side. Excess carriers induced by laser radiation transfer into graphene, making the concentration of electrons and holes to be more balanced, thus leading to the left-hand movement of the Dirac point as shown in Fig. 2d. In the transfer characteristic curve, the intercept on the vertical axis represents the drain current ${I}_{\mathrm{d}}$ of the BPQDs/graphene transistor, As the Dirac point moves left under laser illumination ${I}_{\mathrm{d}}$ decreases, so the photocurrent is negative.

In the process of making BPQDs/graphene on SiO_2_/Si susbstrate, photolithography is performed using a negative tone resist NR9-3000PY. To eliminate the effect of photoresist and to verify our conclusion about the adsorption induced photocurrent, a metal mask is utilized to make the electrodes. Graphene outside of the channel is cut off using a sharp probe on the probe station. By using this method, although the edge of the channel cannot be finely controlled, fundamental mechanisms can be explored easily. The device made by using metal mask is measured in ambient air and vacuum respectively and the result is shown in Fig. 3a. In ambient air, no abrupt change is present, and this result confirms the effect of the photoresist to graphene. In the vacuum environment, the current of BPQDs/graphene device does not recover to the dark current value when the laser is switched off, because the adsorbed water and oxygen is released by the help of incident light. The BPQDs/graphene devices can respond to visible to near-infrared light, as shown in Fig. 3b for the device made by using metal mask. The device is in vacuum and the temperature is 6.9 K. A responsivity of 0.24 mA/W is recorded for the 980 nm laser radiation. The rising speed is relatively fast while the recovery time is long. The response speed is faster than that in ambient environment. The saturation of absorption makes the responsivity of BPQDs/graphene all of the same magnitude for different wavelengths. In the low-temperature environment, the photocurrent is believed to be induced by photoconductivity effect. To exclude the effect of photoresist, the photoactive area is not optimized and our BPQDs/graphene has a lower responsivity and moderate response speed compared to other BPQDs/graphene photodetectors [15].

Photoresist induced photocurrent is first observed to our knowledge. A series of incident laser radiation with different power densities and wavelengths in the visible and infrared range is applied on the graphene exposed to NR9-3000PY photoresist and the photocurrent is shown in Fig. 3c. The polymer molecules in negative tone photoresist cross-link under ultraviolet exposure and form a stable doping at the surface of graphene. The photoresist induced photocurrent is measured for laser wavelength ranging from 405 to 980 nm. The photocurrent clarifies the abrupt change of photocurrent in Fig. 2b. The response time is relatively fast, typically less than one millisecond. The bias-dependent photocurrent under 980 nm laser radiation is shown in Fig. 3d. The rising and falling time is also shown in the inset. The time lag between switch-on of the laser bias and the incident light is considered to be constant and do not interfere with the evaluation of the response speed.

Detailed explanation of charge transfer in the system of adsorbed phosphorus atom or BPQDs and graphene is given by modelling using the first-principle method. BPQDs and graphene are modeled by using an *ab-initio* simulation software OpenMx. Bulk BP possesses an orthorhombic lattice [24–26] and a space group of Cmca [27]. The obtained band structure of monolayer and bulk BP agrees with the results in Ref. [28]. Quantum dots are modeled through making crystal clusters, as done by Chen et al. [29]. The bandgap of BPQDs decreases for a larger diameter of the cluster. As for graphene, the primitive cell of graphene consists of two carbon atoms. The zero bandgap at K point in the Brillouin zone can be easily found, in accordance with the Dirac semi-metal nature of graphene.

**Fig. 4:**
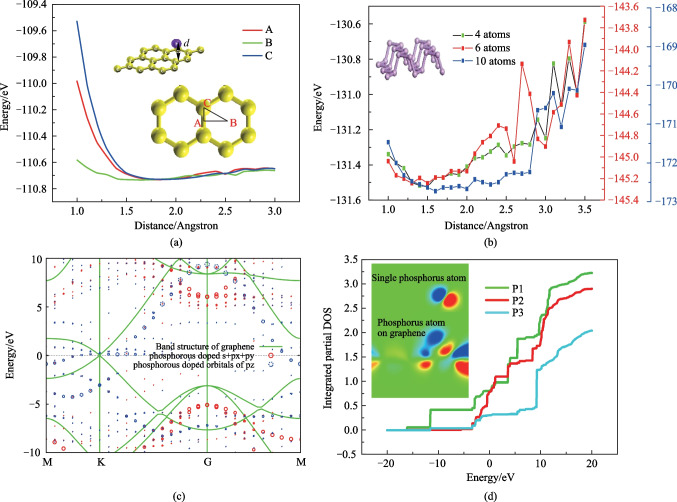
Schematic, energy relaxation and unfolded energy band of phosphorous atoms adsorbed on graphene using the first-principle method. **a** Energy relaxation diagram for phosphorous atom at three positions with high symmetry denoted as A, B and C in the inset figure. **b** Energy relaxation diagram for different size of the BP cluster. **c** Unfolded band structure of a phosphorus atom doping graphene. **d** Integrated partial DOS of three p-orbitals in phosphorus atom adjacent to graphene surface and the inset is HOMO of single phosphorus atom and phosphorus atom absorbed on graphene

The result of phosphorus atoms adsorbed on graphene is given in Fig. 4. When a single phosphorus atom is placed above graphene, as shown in the inset of Fig. 4a, the perpendicular distance between phosphorous atom and graphene plane is denoted as *d*. Due to the symmetry of graphene, it is convenient to find three possible horizontal positions for the phosphorous atom [30–32]. These positions are: in the middle of the C–C bond, at the center of the benzene-like circle, and at a carbon atom, which are denoted as A, B and C respectively. The vertical distance of the phosphorous atom from the graphene plane is changed to find the most stable state. The adsorption energy as a function of distance is shown in Fig. 4a. The lowest total energy for the phosphorous-graphene system is around − 110.7 eV when the phosphorous atom is set at the center of the benzene-like circle, which corresponds to the position B and the green line. In this case, the phosphorous atom has comprehensive connection with all the six carbon atoms around position B, thus stays nearer to the graphene plane. To investigate the energy released in the adsorption process, energy difference is calculated for separated graphene sheet and a single phosphorous atom and the whole system of graphene sheet with an adsorbed phosphorous atom in a stable state. The energy of single phosphorous atom is ${E}_{\mathrm{P}}=-6.7267\;  \mathrm{eV}$, while the energy for graphene sheet is ${E}_{\mathrm{Graphene}}=-103.9401 \; \mathrm{eV}$. The energy for the whole system is ${E}_{\mathrm{total}}=-110.6475 \; \mathrm{eV}$. Thus an energy difference of 0.0193 eV is obtained in this simulation. The connection between the phosphorous and carbon atoms is weaker than an ordinary hydrogen bond. Similar calculations is conducted for the case of BP cluster and the results are shown in Fig. 4b.

To investigate the details of the charge transfer contribution of phosphorus to graphene, we plot the unfolded energy band diagram of a phosphorus atom doping grapheneand the result is presented in Fig. 4c. The dispersion relationship of electron energy is largely enriched around K point by the adsorption of phosphorus atom. For other locations in reciprocal space, the band gaps are reduced to different degrees. Polarization of phosphorus atom after adsorption can be concluded from the integrated partial density of states (PDOS) analysis, as shown in Fig. 4d. Chemical hybridization appears as the phosphorous atom approaches graphene plane. Electrons tend to accumulate near the interface, thus strengthening the p1 and p2 orbitals, leaving the p3 orbital with lower electron density. The highest occupied molecular orbital (HOMO) is shown in the inset of Fig. 4d. The profile of HOMO of phosphorus atom adsorbed on graphene is inclined to carbon atoms. It is found phosphorous atom acts as electron donor and the net charge transfer is − 0.294 e. The calculation is done by transferring the total electron density file obtained in OpenMx to Multiwfn [33], where atom population is calculated based on the Voronoi deformation density (VDD) [34]. The charge transfer calculation is verified by summing net charge difference over all atoms in the system and the result is zero.

## Conclusions

In the BPQDs/graphene device, the introducing of quantum dots reduces the unintentional p-doping of graphene and accounts for the large negative photocurrent. In the ambient air, for the device with BPQDs/graphene on SiO_2_, the total current at zero gate voltage decreases and the Dirac point of graphene moves toward zero with the laser radiation, which means BPQDs anti-dope graphene. The photoresist can play a role in the fast response of light. When the device is in the low temperature in vacuum, phosphorus offers excess carriers and the photocurrent is positive. The BPQDs/graphene device can work in the visible to near-infrared wavelengths at low temperatures. The adsorption effect of BPQDs on graphene is modeled using a first-principle method. The net charge transfer for a phosphorus atom on the graphene is found to be − 0.294 e for the most stable state. A picture of charge transfer and orbital contribution in the interacted BPQDs and single-layer graphene system is also given.

## Data Availability

The data that support the findings of this study are available from the corresponding author, upon reasonable request.
